# 4-Allyl-4-ethyl­morpholinium chloride

**DOI:** 10.1107/S1600536808030201

**Published:** 2008-09-27

**Authors:** Mei-Ling Wang, Hong-Jun Zang, Bo-Wen Cheng

**Affiliations:** a School of Materials and Chemical Engineering and Key Laboratory of Hollow Fiber Membrane Materials & Membrane Processes, Tianjin Polytechnic University, Tianjin 300160, People’s Republic of China

## Abstract

In the title molecular salt, C_9_H_18_NO^+^·Cl^−^, the morpholine ring adopts a chair conformation. In the crystal structure, intra­molecular C—H⋯Cl bonds occur and inter­molecular C—H⋯O and C—H⋯Cl hydrogen bonds link the mol­ecules.

## Related literature

For general background, see: Abedin *et al.* (2004 ▶, 2005 ▶); Kim *et al.* (2005 ▶, 2006 ▶). For bond-length data, see: Allen *et al.* (1987 ▶). For ring puckering parameters, see: Cremer & Pople (1975 ▶).
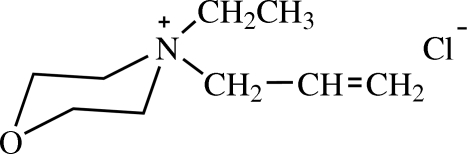


## Experimental

### 

#### Crystal data

C_9_H_18_NO^+^·Cl^−^
*M* *_r_* = 191.69
Monoclinic, 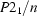

*a* = 8.5414 (17) Å
*b* = 9.0391 (18) Å
*c* = 13.124 (3) Å
β = 91.03 (3)°
*V* = 1013.1 (4) Å^3^
*Z* = 4
Mo *K*α radiation
μ = 0.33 mm^−1^
*T* = 133 (2) K
0.12 × 0.10 × 0.04 mm

#### Data collection

Rigaku Saturn diffractometer
Absorption correction: multi-scan (Jacobson, 1998 ▶) *T*_min_ = 0.961, *T*_max_ = 0.987
5624 measured reflections
1779 independent reflections
1605 reflections with *I* > 2σ(*I*)
*R*_int_ = 0.028

#### Refinement

*R*[*F* ^2^ > 2σ(*F* ^2^)] = 0.029
*wR*(*F* ^2^) = 0.079
*S* = 1.07
1779 reflections
110 parameters
H-atom parameters constrained
Δρ_max_ = 0.23 e Å^−3^
Δρ_min_ = −0.21 e Å^−3^

### 

Data collection: *CrystalClear* (Rigaku/MSC, 2005 ▶); cell refinement: *CrystalClear*; data reduction: *CrystalClear*; program(s) used to solve structure: *SHELXTL* (Sheldrick, 2008 ▶); program(s) used to refine structure: *SHELXTL*; molecular graphics: *SHELXTL* software used to prepare material for publication: *SHELXTL*.

## Supplementary Material

Crystal structure: contains datablocks I, global. DOI: 10.1107/S1600536808030201/hk2524sup1.cif

Structure factors: contains datablocks I. DOI: 10.1107/S1600536808030201/hk2524Isup2.hkl

Additional supplementary materials:  crystallographic information; 3D view; checkCIF report

## Figures and Tables

**Table 1:** Hydrogen-bond geometry (Å, °)

*D*—H⋯*A*	*D*—H	H⋯*A*	*D*⋯*A*	*D*—H⋯*A*
C1—H1*A*⋯O1^i^	0.97	2.48	3.4446 (19)	175
C2—H2*A*⋯Cl1^ii^	0.97	2.69	3.4417 (15)	135
C2—H2*B*⋯Cl1^iii^	0.97	2.72	3.6690 (17)	166
C4—H4*A*⋯Cl1^iv^	0.97	2.83	3.7513 (16)	160
C4—H4*B*⋯Cl1^v^	0.97	2.71	3.5612 (18)	147
C5—H5*A*⋯Cl1^iv^	0.97	2.78	3.6871 (16)	157
C5—H5*B*⋯Cl1^iii^	0.97	2.81	3.7562 (16)	166
C6—H6⋯Cl1	0.93	2.75	3.6777 (18)	173
C7—H7*A*⋯Cl1^iii^	0.93	2.92	3.776 (2)	154
C7—H7*B*⋯O1^vi^	0.93	2.58	3.4456 (19)	155
C9—H9*B*⋯O1^vii^	0.96	2.58	3.5359 (19)	173

Symmetry codes: (i) 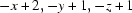; (ii) 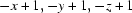; (iii) 
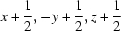
; (iv) 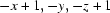; (v) 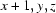; (vi) 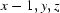; (vii) 
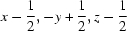
.

**Fig. 1.**
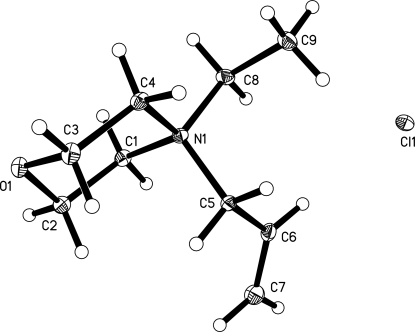
The molecular structure of the title molecule with the atom-numbering scheme. Displacement ellipsoids are drawn at the 30% probability level.

**Fig. 2.**
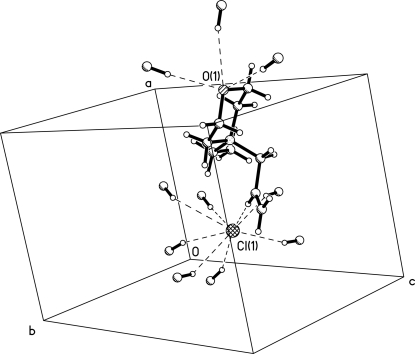
A packing diagram of the title compound. Hydrogen bonds are shown as dashed lines.

## supplementary crystallographic information

### Comment

Quaternary morpholine halides are valuable precursors for the preparation of ionic liquids (ILs) by ion metathesis (Kim *et al.*,  2005). The excellent conductivity, broad electrochemical window, thermal stability, and low volatility of ILs have made them promising media for electrochemical processes (Abedin *et al.*,  2004; Abedin *et al.*,  2005). In particular, ILs based on the morpholinium cation are favored because of their low cost, easy synthesis and electrochemical stability (Kim *et al.*,  2006). So far, only a few crystallographic studies have been performed on salts. We report herein the crystal structure of the title compound.

In the molecule of the title compound, (Fig. 1), the bond lengths (Allen *et al.*,  1987) and angles are generally within normal ranges. The morpholine ring (O1/N1/C1-C4) is, of course, not planar, having total puckering amplitude, Q_T_, of 1.085 (3) and chair conformation [φ = -154.63 (3)° and θ = 122.70 (3)°] (Cremer & Pople, 1975).

In the crystal structure, intramolecular C-H···Cl and intermolecular C-H···O and C-H···Cl hydrogen bonds (Table 1) link the molecules (Fig. 2), in which they may be effective in the stabilization of the structure.

### Experimental

Under vigorous stirring, allyl chloride (0.1 mol) was added to a solution of 4-ethylmorpholine (0.1 mol) in acetonitrile (20 ml). The mixture was stirred at 333 K for 2 h. The mixture was filtered to remove excess *N*-ethyl morpholine and allyl chloride and washed with acetone to give the title compound. It was crystallized from ethanol/acetone mixture (1:20) by slow evaporation.

### Refinement

H atoms were positioned geometrically, with C-H = 0.93, 0.97 and 0.96 Å for aromatic, methylene and methyl H, respectively, and constrained to ride on their parent atoms with U_iso_(H) = xU_eq_(C), where x = 1.5 for methyl H and x = 1.2 for all other H atoms.

### Crystal data

**Table:**

C_9_H_18_NO^+^·Cl^−^	*F*(000) = 416
*M**_r_* = 191.69	*D*_x_ = 1.257 Mg m^−^^3^
Monoclinic, *P*2_1_/*n*	Mo *K*α radiation, λ = 0.71073 Å
Hall symbol: -P 2yn	Cell parameters from 1775 reflections
*a* = 8.5414 (17) Å	θ = 2.1–27.8°
*b* = 9.0391 (18) Å	µ = 0.33 mm^−^^1^
*c* = 13.124 (3) Å	*T* = 133 K
β = 91.03 (3)°	Prism, colorless
*V* = 1013.1 (4) Å^3^	0.12 × 0.10 × 0.04 mm
*Z* = 4	

### Data collection

**Table:**

Rigaku Saturn diffractometer	1779 independent reflections
Radiation source: fine-focus sealed tube	1605 reflections with *I* > 2σ(*I*)
confocal	*R*_int_ = 0.028
Detector resolution: 27.571 pixels mm^-1^	θ_max_ = 25.0°, θ_min_ = 2.7°
ω scans	*h* = −10→10
Absorption correction: multi-scan (Jacobson, 1998)	*k* = −10→10
*T*_min_ = 0.961, *T*_max_ = 0.987	*l* = −15→7
5624 measured reflections	

### Refinement

**Table:**

Refinement on *F*^2^	Primary atom site location: structure-invariant direct methods
Least-squares matrix: full	Secondary atom site location: difference Fourier map
*R*[*F*^2^ > 2σ(*F*^2^)] = 0.029	Hydrogen site location: inferred from neighbouring sites
*wR*(*F*^2^) = 0.079	H-atom parameters constrained
*S* = 1.08	*w* = 1/[σ^2^(*F*_o_^2^) + (0.0427*P*)^2^ + 0.2964*P*] where *P* = (*F*_o_^2^ + 2*F*_c_^2^)/3
1779 reflections	(Δ/σ)_max_ < 0.001
110 parameters	Δρ_max_ = 0.24 e Å^−^^3^
0 restraints	Δρ_min_ = −0.21 e Å^−^^3^

### Special details

**Table:**

Geometry. All e.s.d.'s (except the e.s.d. in the dihedral angle between two l.s. planes) are estimated using the full covariance matrix. The cell e.s.d.'s are taken into account individually in the estimation of e.s.d.'s in distances, angles and torsion angles; correlations between e.s.d.'s in cell parameters are only used when they are defined by crystal symmetry. An approximate (isotropic) treatment of cell e.s.d.'s is used for estimating e.s.d.'s involving l.s. planes.
Refinement. Refinement of *F*^2^ against ALL reflections. The weighted *R*-factor *wR* and goodness of fit *S* are based on *F*^2^, conventional *R*-factors *R* are based on *F*, with *F* set to zero for negative *F*^2^. The threshold expression of *F*^2^ > σ(*F*^2^) is used only for calculating *R*-factors(gt) *etc*. and is not relevant to the choice of reflections for refinement. *R*-factors based on *F*^2^ are statistically about twice as large as those based on *F*, and *R*- factors based on ALL data will be even larger.

### Fractional atomic coordinates and isotropic or equivalent isotropic displacement parameters (Å^2^)

**Table:**

	*x*	*y*	*z*	*U*_iso_*/*U*_eq_	
Cl1	0.25140 (4)	0.17741 (4)	0.39587 (3)	0.01833 (14)	
O1	1.03933 (11)	0.39901 (11)	0.64961 (8)	0.0178 (2)	
N1	0.76318 (13)	0.27908 (13)	0.54538 (9)	0.0132 (3)	
C1	0.79277 (17)	0.44310 (15)	0.56086 (11)	0.0152 (3)	
H1A	0.8448	0.4824	0.5016	0.018*	
H1B	0.6932	0.4937	0.5668	0.018*	
C2	0.89224 (17)	0.47404 (15)	0.65487 (11)	0.0169 (3)	
H2A	0.9101	0.5797	0.6609	0.020*	
H2B	0.8373	0.4415	0.7149	0.020*	
C3	1.01221 (17)	0.24347 (15)	0.64773 (11)	0.0177 (3)	
H3A	0.9565	0.2149	0.7084	0.021*	
H3B	1.1119	0.1920	0.6484	0.021*	
C4	0.91803 (16)	0.19804 (15)	0.55435 (11)	0.0164 (3)	
H4A	0.8983	0.0924	0.5571	0.020*	
H4B	0.9787	0.2177	0.4941	0.020*	
C5	0.65242 (16)	0.21894 (15)	0.62513 (11)	0.0140 (3)	
H5A	0.6438	0.1126	0.6169	0.017*	
H5B	0.6970	0.2378	0.6924	0.017*	
C6	0.49194 (17)	0.28520 (16)	0.61896 (11)	0.0177 (3)	
H6	0.4292	0.2668	0.5617	0.021*	
C7	0.43728 (18)	0.36865 (18)	0.69216 (12)	0.0242 (4)	
H7A	0.4988	0.3880	0.7498	0.029*	
H7B	0.3371	0.4084	0.6863	0.029*	
C8	0.69654 (17)	0.25945 (16)	0.43820 (11)	0.0177 (3)	
H8A	0.5981	0.3127	0.4328	0.021*	
H8B	0.7680	0.3041	0.3905	0.021*	
C9	0.66857 (19)	0.10082 (16)	0.40710 (11)	0.0220 (4)	
H9A	0.7662	0.0481	0.4085	0.033*	
H9B	0.6241	0.0980	0.3394	0.033*	
H9C	0.5976	0.0554	0.4536	0.033*	

### Atomic displacement parameters (Å^2^)

**Table:**

	*U*^11^	*U*^22^	*U*^33^	*U*^12^	*U*^13^	*U*^23^
Cl1	0.0215 (2)	0.0166 (2)	0.0169 (2)	0.00409 (13)	−0.00052 (15)	−0.00080 (13)
O1	0.0150 (6)	0.0159 (5)	0.0223 (6)	−0.0008 (4)	−0.0006 (4)	−0.0002 (4)
N1	0.0141 (6)	0.0130 (6)	0.0126 (6)	0.0005 (5)	0.0015 (5)	0.0012 (5)
C1	0.0168 (7)	0.0107 (7)	0.0182 (7)	0.0006 (5)	0.0024 (6)	0.0021 (6)
C2	0.0179 (8)	0.0136 (7)	0.0192 (7)	0.0007 (6)	0.0020 (6)	−0.0005 (6)
C3	0.0156 (7)	0.0141 (7)	0.0234 (8)	0.0024 (5)	−0.0008 (6)	0.0013 (6)
C4	0.0137 (8)	0.0151 (7)	0.0204 (8)	0.0034 (5)	0.0028 (6)	−0.0006 (6)
C5	0.0154 (7)	0.0138 (6)	0.0130 (7)	−0.0020 (5)	0.0021 (6)	0.0009 (6)
C6	0.0138 (7)	0.0213 (7)	0.0179 (8)	−0.0033 (6)	−0.0012 (6)	0.0027 (6)
C7	0.0171 (8)	0.0290 (8)	0.0263 (9)	0.0036 (6)	0.0008 (7)	−0.0017 (7)
C8	0.0199 (8)	0.0213 (8)	0.0118 (7)	0.0011 (6)	−0.0003 (6)	0.0013 (6)
C9	0.0256 (9)	0.0241 (8)	0.0161 (8)	−0.0025 (6)	−0.0018 (6)	−0.0025 (6)

### Geometric parameters (Å, °)

**Table:**

O1—C2	1.4305 (17)	C4—H4B	0.9700
O1—C3	1.4250 (17)	C5—H5A	0.9700
N1—C1	1.5170 (17)	C5—H5B	0.9700
N1—C4	1.5146 (17)	C6—C5	1.497 (2)
N1—C5	1.5237 (18)	C6—C7	1.314 (2)
N1—C8	1.5183 (18)	C6—H6	0.9300
C1—C2	1.511 (2)	C7—H7A	0.9300
C1—H1A	0.9700	C7—H7B	0.9300
C1—H1B	0.9700	C8—C9	1.509 (2)
C2—H2A	0.9700	C8—H8A	0.9700
C2—H2B	0.9700	C8—H8B	0.9700
C3—C4	1.511 (2)	C9—H9A	0.9600
C3—H3A	0.9700	C9—H9B	0.9600
C3—H3B	0.9700	C9—H9C	0.9600
C4—H4A	0.9700		
			
C3—O1—C2	109.02 (10)	C3—C4—H4A	109.1
C1—N1—C5	111.15 (11)	C3—C4—H4B	109.1
C1—N1—C8	107.28 (10)	H4A—C4—H4B	107.8
C4—N1—C1	108.61 (10)	N1—C5—H5A	108.9
C4—N1—C5	109.04 (11)	N1—C5—H5B	108.9
C4—N1—C8	109.13 (11)	C6—C5—N1	113.54 (11)
C8—N1—C5	111.57 (11)	C6—C5—H5A	108.9
N1—C1—H1A	109.1	C6—C5—H5B	108.9
N1—C1—H1B	109.1	H5A—C5—H5B	107.7
H1A—C1—H1B	107.9	C7—C6—C5	121.82 (14)
C2—C1—N1	112.33 (11)	C7—C6—H6	119.1
C2—C1—H1A	109.1	C5—C6—H6	119.1
C2—C1—H1B	109.1	C6—C7—H7A	120.0
O1—C2—C1	110.74 (12)	C6—C7—H7B	120.0
O1—C2—H2A	109.5	H7A—C7—H7B	120.0
O1—C2—H2B	109.5	N1—C8—H8A	108.6
C1—C2—H2A	109.5	N1—C8—H8B	108.6
C1—C2—H2B	109.5	C9—C8—N1	114.62 (11)
H2A—C2—H2B	108.1	C9—C8—H8A	108.6
O1—C3—C4	111.49 (11)	C9—C8—H8B	108.6
O1—C3—H3A	109.3	H8A—C8—H8B	107.6
O1—C3—H3B	109.3	C8—C9—H9A	109.5
C4—C3—H3A	109.3	C8—C9—H9B	109.5
C4—C3—H3B	109.3	H9A—C9—H9B	109.5
H3A—C3—H3B	108.0	C8—C9—H9C	109.5
N1—C4—H4A	109.1	H9A—C9—H9C	109.5
N1—C4—H4B	109.1	H9B—C9—H9C	109.5
C3—C4—N1	112.52 (11)		
			
N1—C1—C2—O1	57.87 (15)	C1—N1—C5—C6	63.78 (14)
C3—O1—C2—C1	−63.03 (14)	C4—N1—C5—C6	−176.51 (11)
C2—O1—C3—C4	62.46 (15)	C8—N1—C5—C6	−55.91 (15)
C4—N1—C1—C2	−49.24 (15)	C1—N1—C8—C9	176.57 (12)
C5—N1—C1—C2	70.72 (14)	C4—N1—C8—C9	59.07 (15)
C8—N1—C1—C2	−167.07 (12)	C5—N1—C8—C9	−61.48 (15)
C1—N1—C4—C3	48.38 (15)	O1—C3—C4—N1	−56.48 (16)
C5—N1—C4—C3	−72.88 (14)	C7—C6—C5—N1	−114.26 (16)
C8—N1—C4—C3	165.03 (11)		

### Hydrogen-bond geometry (Å, °)

**Table:**

*D*—H···*A*	*D*—H	H···*A*	*D*···*A*	*D*—H···*A*
C1—H1A···O1^i^	0.97	2.48	3.4446 (19)	175.
C2—H2A···Cl1^ii^	0.97	2.69	3.4417 (15)	135.
C2—H2B···Cl1^iii^	0.97	2.72	3.6690 (17)	166.
C4—H4A···Cl1^iv^	0.97	2.83	3.7513 (16)	160.
C4—H4B···Cl1^v^	0.97	2.71	3.5612 (18)	147.
C5—H5A···Cl1^iv^	0.97	2.78	3.6871 (16)	157.
C5—H5B···Cl1^iii^	0.97	2.81	3.7562 (16)	166.
C6—H6···Cl1	0.93	2.75	3.6777 (18)	173.
C7—H7A···Cl1^iii^	0.93	2.92	3.776 (2)	154.
C7—H7B···O1^vi^	0.93	2.58	3.4456 (19)	155.
C9—H9B···O1^vii^	0.96	2.58	3.5359 (19)	173.

Symmetry codes: (i) −*x*+2, −*y*+1, −*z*+1; (ii) −*x*+1, −*y*+1, −*z*+1; (iii) *x*+1/2, −*y*+1/2, *z*+1/2; (iv) −*x*+1, −*y*, −*z*+1; (v) *x*+1, *y*, *z*; (vi) *x*−1, *y*, *z*; (vii) *x*−1/2, −*y*+1/2, *z*−1/2.
